# Sinus bradycardia as an atypical presentation of severe hypercalcemia: a case report and a literature review

**DOI:** 10.1097/MS9.0000000000004802

**Published:** 2026-02-20

**Authors:** Besher Shami, Riyadh Saif, Iman Mousselli, Souhila Belhandouz, Hafsa Ashraf, Katherin Zambrano, Mostafa Helou, Kalpana Chilukuri, Pradeep Manoharan

**Affiliations:** a Department of Medicine, University of Massachusetts, Berkshire Medical Center, Pittsfield, Massachusetts, USA; b Department of Medicine, University of Aleppo, Faculty of Medicine, Aleppo, Syria

**Keywords:** arrhythmia, case report, hypercalcemia-induced bradycardia, paraneoplastic

## Abstract

**Introduction and importance:**

Severe hypercalcemia typically produces QT interval shortening or ventricular arrhythmias, whereas bradyarrhythmias are exceptionally rare. The coexistence of hypercalcemia with acute kidney injury (AKI) – a condition that usually causes hypocalcemia – further underscores diagnostic complexity.

**Case presentation:**

An 85-year-old man presented with AKI, confusion, and symptomatic sinus bradycardia (40 bpm) without atrioventricular block or QT prolongation. Laboratory studies showed markedly elevated corrected calcium, suppressed parathyroid hormone, and increased parathyroid hormone–related peptide. Chest CT revealed a 7 × 8 mm right-lung nodule. These findings are suggestive of humoral hypercalcemia of malignancy, although the etiology remains unconfirmed in the absence of tissue diagnosis. Treatment with intravenous hydration and zoledronic acid led to normalization of calcium, restoration of sinus rhythm (61 bpm), and improvement of renal function, obviating the need for pacing.

**Discussion:**

This case is distinctive for demonstrating reversible sinus bradycardia in the setting of severe hypercalcemia and acute kidney injury – an atypical and physiologically unexpected combination. The biochemical profile raises suspicion for a paraneoplastic process, though this remains unconfirmed. The case underscores the importance of recognizing hypercalcemia as a reversible cause of bradyarrhythmia and considering malignancy in the appropriate clinical context when PTH is suppressed.

**Conclusion:**

Hypercalcemia-induced bradycardia, though rare, is fully reversible with prompt metabolic correction. Awareness of this atypical presentation can prevent misdiagnosis, unnecessary pacemaker implantation, and delayed recognition of paraneoplastic disease.

## Introduction

Electrolyte imbalances are well-recognized precipitants of cardiac arrhythmias. Among them, hypercalcemia is relatively uncommon, with an estimated prevalence of 1–2% in the general population and up to 2% of hospitalized patients^[1,2]^. Most cases result from primary hyperparathyroidism or malignancy-associated hypercalcemia, while less common causes include vitamin D intoxication, granulomatous disease, medications, and other endocrine disorders^[3–5]^.

The cardiovascular effects of hypercalcemia are classically characterized by QT interval shortening, the presence of Osborn (J) waves, and, in severe cases, ventricular arrhythmias^[6,7]^. Bradyarrhythmias are infrequently encountered, and their mechanisms remain incompletely understood. Proposed explanations include impaired sinoatrial node automaticity, slowed atrioventricular (AV) nodal conduction, and calcium-mediated modulation of sodium channel activity^[7,8]^. Recognition of this rare presentation is clinically important, as correction of hypercalcemia may rapidly reverse bradyarrhythmias and prevent unnecessary interventions such as pacemaker implantation.

**HIGHLIGHTS**

- Hypercalcemia can rarely cause clinically significant bradyarrhythmia.
- Correction of calcium levels may rapidly restore normal sinus rhythm.
- Recognition prevents unnecessary pacemaker implantation in elderly patients.
- Hypercalcemia with suppressed PTH should prompt evaluation for malignancy.

The relationship between calcium balance and renal function adds further diagnostic complexity. In acute kidney injury (AKI) and chronic kidney disease (CKD), the expected biochemical profile is hypocalcemia due to phosphate retention and impaired calcitriol synthesis^[9]^. Therefore, hypercalcemia in the setting of renal dysfunction should prompt evaluation for alternative etiologies, particularly paraneoplastic syndromes. In such cases, suppressed parathyroid hormone (PTH) with elevated parathyroid hormone–related peptide (PTHrP) strongly suggests humoral hypercalcemia of malignancy (HHM)^[3,10,11]^.

We describe the case of an 85-year-old man admitted with AKI who developed symptomatic sinus bradycardia in the context of severe hypercalcemia, suppressed PTH, and mildly elevated PTHrP. This report underscores the importance of considering hypercalcemia as a reversible cause of bradyarrhythmia and highlights diagnostic challenges at the intersection of nephrology, endocrinology, and cardiology.

Our article is compliant with the TITAN Guidelines 2025^[12]^.

## Case presentation

An 85-year-old male with a history of coronary artery disease (CAD), hypertension, chronic obstructive pulmonary disease (COPD), Barrett’s esophagus, and chronic opioid use presented with 2 days of nausea, vomiting, constipation, abdominal pain, progressive weakness, confusion, and an unintentional 60-pound weight loss over 6 months. Medications include Atorvastatin, Clopidogrel, Famotidine, Lisinopril, and Oxycodone.

On examination, he was a frail elderly male with vital signs as follows: blood pressure 185/69 mm Hg, pulse 55 bpm, respiratory rate 16 breaths/min, temperature afebrile. Cardiac examination revealed a regular rhythm with mild bradycardia. Lungs were clear to auscultation, and the abdomen was mildly tender with decreased bowel sounds.

Laboratory testing showed anemia with hemoglobin 9.2 g/dl (normal 12–16) and hematocrit 28.6% (normal 37–50%), with normal white blood cell and platelet counts. Serum creatinine was 2.57 mg/dl (baseline ~ 1.3 mg/dl), BUN 57 mg/dl (normal 9–20), and GFR 24 ml/min (baseline >60), consistent with AKI. Potassium and calcium were elevated at 5.4 mEq/l (normal 3.5–5.1) and 11.5 mg/dl (normal 8.5–10.5), respectively.

CT scan of the kidneys revealed no hydronephrosis or obstruction, only simple right-sided cortical cysts. The AKI was suspected to be prerenal, secondary to gastrointestinal losses in the setting of ACE-inhibitor therapy, and IV fluids were initiated.

**Figure 1.**
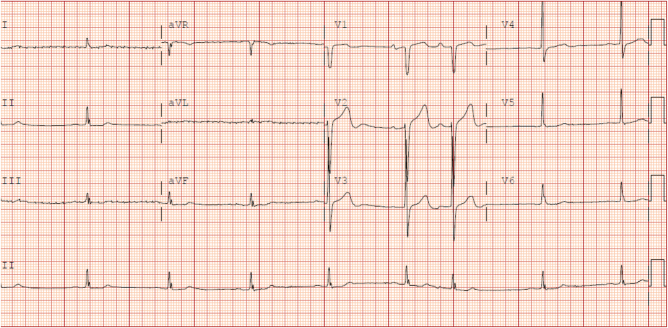
ECG on Day 0 revealing sinus bradycardia (HR = 40) with otherwise normal PR, QRS, and QTc intervals – no AV block or conduction delay.

During hospitalization (Day 0), the patient developed dizziness and lightheadedness. ECG showed sinus bradycardia with each QRS complex preceded by a P wave and a ventricular rate of approximately 40 bpm, normal PR interval (≈0.16–0.18 s), normal QRS duration (≈0.08–0.09 s), with QT interval appeared prolonged relative to the slow rate; however, the corrected QT (QTc) by Bazett’s formula was approximately 400–420 ms, which remains within normal limits (Fig. 1).

At that time, laboratory testing revealed corrected calcium 13.2 mg/dl, suppressed PTH 13 pg/ml (normal 18–88), elevated PTHrP 31 pmol/l (normal <2), phosphate 3.6 mg/dl, 25-hydroxyvitamin D 86 ng/ml, 1,25-dihydroxyvitamin D 96 pg/ml, and low total protein 5.1 g/dl on serum protein electrophoresis. Troponin was normal at 20 ng/l.

Laboratory findings are summarized in Table 1.

**Table 1:** Presentation vs discharge labs

Laboratory test	On presentation	On discharge	Reference range
Corrected Calcium (mg/dl)	13.2	9.5	8.5–10.5
Creatinine (mg/dl)	2.57	1.29	0.6–1.3
BUN (mg/dl)	57.0	33.0	7–20
Troponin (ng/l)	20	21	0–53
Phosphate (mg/dl)	3.6	3.8	2.5–4.5
PTH (pg/ml)	13.0	19.0	15–65
Albumin (g/dl)	2.7	3.0	3.5–5.0
PTHrP (pmol/l)	31.0	–	< 2.0
25-OH Vitamin D (ng/ml)	86.0	–	20–50
1,25-OH Vitamin D (pg/ml)	96.0	–	18–72

Laboratory trends demonstrating resolution of hypercalcemia and improvement in renal function following therapy. The markedly elevated corrected calcium and suppressed PTH on admission, together with elevated PTHrP, were consistent with humoral hypercalcemia of malignancy (HHM). With intravenous hydration and bisphosphonate therapy, calcium and creatinine normalized by discharge.

A comprehensive differential diagnosis for bradyarrhythmia was considered. Medication-induced bradycardia was excluded, as the patient was not taking beta-blockers, calcium channel blockers, digoxin, or other negative chronotropes; although he was on chronic opioid therapy, opioid-induced bradycardia is rare, typically transient, and does not account for the concurrent metabolic derangements observed, with no recent changes in the patient’s daily doses. Medication review also excluded calcium supplements or antacids, ruling out milk-alkali syndrome. Table 2 describes patient’s medications list with doses and last intake. Ischemic heart disease was ruled out by the absence of chest pain, ischemic ST-T changes on ECG, and normal cardiac troponin levels. Primary conduction system disease was considered less likely, given the temporal association between elevated calcium levels and bradycardia, and the complete resolution of rhythm abnormality following calcium normalization. Endocrine causes, including hypothyroidism and adrenal insufficiency, were excluded by normal thyroid-stimulating hormone (1.11 μIU/ml) and morning cortisol levels (17 mcg/dl). Normal PTH excluded hyperparathyroidism, while normal vitamin D levels ruled out vitamin D–related causes. Other metabolic causes were excluded with normal electrolytes (K 4.9 mEq/l, Mg 1.6 mg/dl), Infectious etiologies, such as Lyme disease, were also ruled out with a negative tick-borne panel. Hypothermia was ruled out with normal body temperature, and patient has no family history of hypercalcemia. Collectively, these findings supported hypercalcemia as the sole and reversible cause of sinus bradycardia in this patient.

**Table 2:** Home medications with corresponding doses, routes, and timing of last intake

Medication	Dose/Formulation	Route	Last Intake	Notes/Relevance
Albuterol sulfate	90 mcg per actuation (inhaler)	Inhalation	–	For COPD; no known bradycardic effect
Atorvastatin	40 mg tablet	Oral	Morning of first bradycardia episode	Lipid-lowering agent; not associated with bradycardia
Breo Ellipta	200/25 mcg (fluticasone furoate/vilanterol) inhaler	Inhalation	–	Long-acting β_2_-agonist and corticosteroid; unlikely contributor
Clopidogrel	75 mg tablet	Oral	Morning of first bradycardia episode	Antiplatelet; no bradycardic effect
Famotidine	40 mg tablet	Oral	Morning of first bradycardia episode	H_2_-blocker; no bradycardic effect
Lisinopril	20 mg tablet	Oral	Before admission. Was held due to AKI	ACE inhibitor; may contribute indirectly via AKI, but not bradycardia
Oxycodone	20 mg tablet	Oral	Morning of first bradycardia episode	Chronic use; opioid-induced bradycardia considered but unlikely primary cause

None of the listed medications were deemed causal for the patient’s bradycardia.

**Figure 2.**
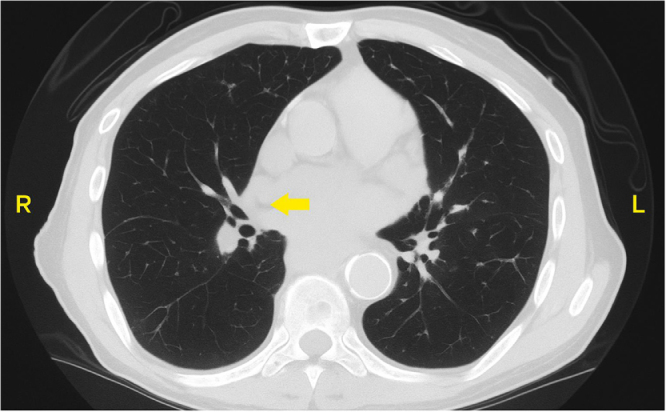
Chest CT with an irregular 7 × 8 mm pulmonary nodule along the right minor fissure.

Chest CT demonstrated an irregular 7 × 8 mm pulmonary nodule along the right minor fissure (Fig. 2). Oncology consultation determined that the nodule was too small for biopsy and recommended outpatient PET scan and follow-up CT in 6 months. The working diagnosis was symptomatic hypercalcemia with a biochemical pattern suggestive of a paraneoplastic process, although this remains unconfirmed, as no tissue diagnosis has been obtained.

The patient was treated with IV hydration with normal saline for a total of 4 liters on a rate of 100 ml/hour, and a single dose of IV zoledronic acid 4 mg given on Day 2. Although loop diuretics can be effective for hypercalcemia treatment, they were not considered in our case due to patient clinical status with AKI and clinical volume depletion. Modern guidelines emphasize that loop diuretics add little benefit unless fluid overload is present. A discussion was also held regarding the potential use of calcitonin; however, this option was deferred because of its short duration of action and limited efficacy, as it typically reduces serum calcium by only 1–2 mg/dl compared with the more potent and sustained effects of bisphosphonates or denosumab. Denosumab was likewise not administered, as the patient’s renal dysfunction was prerenal and reversible, permitting the safe use of intravenous zoledronic acid. Denosumab is generally reserved for cases of bisphosphonate-refractory hypercalcemia or severe chronic kidney disease, neither of which applied in this scenario.

Renal function was closely monitored throughout bisphosphonate therapy given the patient’s baseline acute kidney injury. Serum creatinine, blood urea nitrogen, and urine output were assessed daily during hospitalization. No acute deterioration in renal indices occurred following infusion; in fact, renal parameters showed steady improvement, with progressive reductions in serum creatinine and BUN levels, while the patient maintained an average urine output of approximately 2 liters per day throughout hospitalization. This trend supported both the reversibility of the prerenal injury and the safe renal tolerability of bisphosphonate therapy in this case.

Continuous telemetry through hospitalization confirmed persistent sinus bradycardia without pauses, AV block, sinus arrest, or additional arrhythmias. Additionally, no episodes of syncope or hypotension were noted. Therefore, emergent pharmacologic or pacing interventions were not required. Specifically, atropine and isoproterenol were withheld, as there was no evidence of unstable bradyarrhythmia warranting acute chronotropic support. Likewise, temporary transvenous pacing was not indicated because the bradycardia was sinus in origin, not due to conduction block, and was expected to improve with correction of the underlying metabolic abnormality.

**Figure 3.**
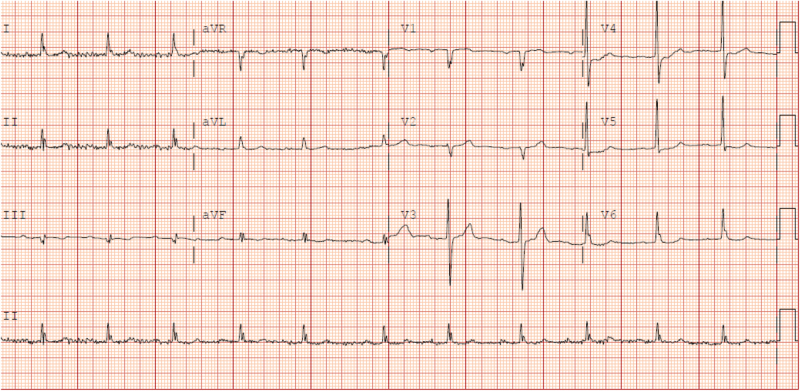
ECG on discharge with normal sinus rhythm at 61 bpm with normal PR, QRS, and QTc intervals. Conduction is intact, and the previously observed bradycardia has resolved.

**Figure 4.**
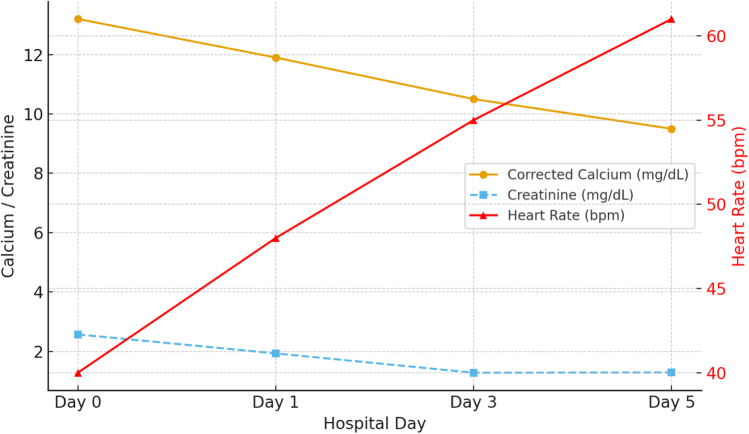
Trends in calcium, Cr, and HR during hospitalization.

Calcium levels decreased progressively from 13.2 mg/dl to 11.9 mg/dl and finally to 9.5 mg/dl at discharge. Heart rate improved in parallel, rising from 40 bpm at admission to 61 bpm, and the ECG on discharged demonstrated a normal sinus rhythm with each QRS complex preceded by a P wave, normal PR interval measures around 0.16 seconds (160 ms), and normal QRS duration at approximately 0.08 seconds (80 ms), normal QT interval i, with a corrected QT (QTc) of roughly 410–420 ms (Fig. 3). Gastrointestinal and neurocognitive symptoms resolved, and renal function recovered (creatinine improved from 2.57 to 1.29 mg/dl; Fig. 4). The patient was discharged in stable condition with outpatient oncology follow-up.

**Figure 5.**
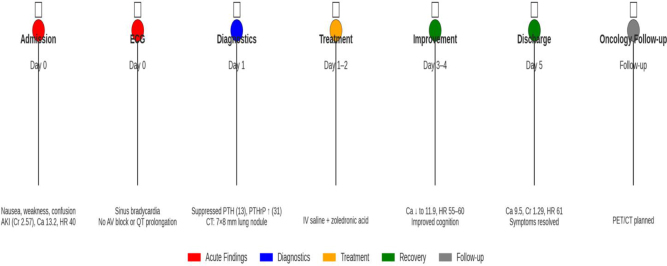
A visual timeline summary for the case.

Figure 5 describes a brief visual timeline for the case.

## Discussion

Hypercalcemia is most often associated with QT interval shortening, atrial and ventricular ectopy, and, in severe cases, ventricular arrhythmias^[6,7]^. Bradyarrhythmias are rare, and most reported cases involve sinus bradycardia or varying degrees of heart block. In our patient, symptomatic sinus bradycardia resolved with correction of hypercalcemia, suggesting a causal relationship.

The electrophysiologic effects of hypercalcemia on cardiac conduction are multifactorial. Elevated extracellular calcium increases threshold potential and stabilizes cardiac cell membranes, thereby reducing myocardial excitability and automaticity. In the sinoatrial (SA) node, excess calcium blunts phase 4 depolarization by suppressing inward sodium and calcium currents, leading to slowed impulse generation and sinus bradycardia. At the atrioventricular (AV) node, hypercalcemia can prolong conduction time and increase refractoriness, predisposing to varying degrees of heart block. Experimental studies also suggest partial inactivation of fast sodium channels, slowing conduction velocity throughout the His-Purkinje system. Additionally, high calcium levels shorten ventricular action-potential duration and QT interval by accelerating phase 2 repolarization, which may further impair coordinated conduction and promote arrhythmogenesis. These combined effects explain the spectrum of rhythm disturbances observed in severe hypercalcemia – from sinus bradycardia and junctional rhythms to complete AV block – and underscore the reversibility of these abnormalities once serum calcium normalizes^[3,13,14]^.

In the setting of acute kidney injury (AKI), hypercalcemia is physiologically unexpected because renal dysfunction generally causes hypocalcemia through phosphate retention, reduced 1-α hydroxylation of vitamin D, and decreased intestinal calcium absorption^[15]^. When hypercalcemia occurs in AKI, it should prompt careful evaluation for alternative or concurrent mechanisms such as malignancy, excessive calcium or vitamin D intake, tertiary hyperparathyroidism, or laboratory artifact. In this patient, hypercalcemia was true and reproducible, confirmed on repeat testing, and accompanied by suppressed parathyroid hormone (PTH) and elevated parathyroid hormone–related peptide (PTHrP), *suggestive* of humoral hypercalcemia of malignancy (HHM). However, because no tissue diagnosis is available, malignancy cannot be confirmed. The pulmonary nodule raises suspicion but remains an unproven source pending additional evaluation.

Scattered case reports have described sinus-node dysfunction, junctional rhythms, and varying degrees of AV block, most often associated with primary hyperparathyroidism (PHPT) and less commonly with malignancy-related hypercalcemia. Shah *et al* first highlighted sinus-node dysfunction secondary to hypercalcemia from PHPT, emphasizing that only a handful of such reports existed at the time^[8]^. Akinseye *et al* reported severe hypercalcemia resulting in sinus arrest that reversed following calcium normalization^[16]^. Gutiérrez and Ramakumar described complete heart block secondary to hypercalcemia that resolved following treatment^[17,18]^, while Vosnakidis *et al* described reversible AV block following normalization of calcium levels^[19]^. Thotakura *et al* reported complete AV block at a corrected calcium of 17.8 mg/dl due to paraneoplastic PTHrP-mediated hypercalcemia, which resolved after calcium correction^[20]^. Similarly, other reports confirmed that hypercalcemia can cause sinus bradycardia, high-grade AV block, or sinus arrest, though these presentations remain uncommon^[21–23]^. Across these reports, AV block and severe conduction abnormalities typically occurred at calcium levels exceeding 15 mg/dl, whereas isolated sinus bradycardia was observed at lower – though still markedly elevated concentrations. Our case adds to this limited evidence by describing isolated sinus bradycardia at a corrected calcium of 13.2 mg/dl due to PTHrP-mediated HHM – a distinctly uncommon presentation compared with PHPT-related cases. This highlights the importance of recognizing hypercalcemia as a potentially reversible cause of bradyarrhythmia, preventing unnecessary pacemaker implantation, and emphasizing prompt metabolic correction as the cornerstone of management.

From a cardiovascular standpoint, prompt correction of hypercalcemia is critical to reversing conduction abnormalities and preventing unnecessary pacemaker implantation. Recognizing hypercalcemia as a reversible cause of bradyarrhythmia carries major clinical implications. In hospitalized or older patients, bradycardia or atrioventricular (AV) block often triggers consideration for permanent pacemaker placement. However, early pacing without exclusion of reversible metabolic or pharmacologic causes exposes patients to avoidable procedural risks, device-related infections, long-term lead complications, and unnecessary lifelong follow-up. Multiple case reports, including those summarized in Table 3, have documented complete normalization of sinus or AV nodal function after calcium correction, confirming that conduction abnormalities can be transient rather than structural. Before proceeding to pacing, clinicians should perform a thorough evaluation of metabolic, endocrine, and medication-related factors that may depress nodal automaticity. Temporary pacing may be warranted only in cases of hemodynamic instability that persist despite initial corrective therapy. In our patient, prompt treatment of hypercalcemia led to recovery of normal sinus rhythm within 24 hours, eliminating the need for permanent pacing.

**Table 3:** Published cases of hypercalcemia-related brady-arrhythmias

Author, year	Arrhythmia type	Peak or corrected calcium (mg/dl)	Etiology	Outcome after correction
Shah AP *et al*, 2004 (*Pacing Clin Electrophysiol*)	Sinus-node dysfunction/marked sinus bradycardia	≈ 14–15 (total)	Primary hyperparathyroidism	Heart rate normalized after calcium normalization
Akinseye OA *et al*, 2015 (*Am J Med Case Rep*)	Sinus arrest	15.9 (total)	Primary hyperparathyroidism	Sinus rhythm restored following calcium correction
Gutiérrez JA *et al*, 2016 (*Tex Heart Inst J*)	Complete heart block	15.7 (total)	Primary hyperparathyroidism	Conduction normalized after calcium correction
Ramakumar V *et al*, 2021 (*Cureus*)	Complete heart block	15.5 (total)	Primary hyperparathyroidism	Conduction normalized following Ca correction
Vosnakidis A *et al*, 2013 (*Hellenic J Cardiol*)	Intermittent 2:1 AV block	14.8 (total)	Primary hyperparathyroidism	AV conduction recovered; no pacemaker required
Thotakura S *et al*, 2016 (*Federal Practitioner*)	Complete (3°) AV block, HR 29 bpm, QTc 556 ms	17.5–17.8 (corrected)	PTHrP-mediated HHM	AV block resolved as Ca fell to 9.6 mg/dl
Kelwade JV *et al*, 2016 (*J Assoc Physicians India*)	Sinus arrest/severe sinus bradycardia	16.2 (total)	Primary hyperparathyroidism	Sinus rhythm restored post-parathyroidectomy
Abu-Abaa M *et al*, 2022 (*Eur J Case Rep Intern Med*)	Sinus bradycardia (HR 40 bpm)	14.0 (total)	Primary hyperparathyroidism	Pacemaker avoided; rhythm normalized
Khederlou H *et al*, 2023 (*Eur Heart J Case Rep*)	Complete heart block (paraneoplastic)	16.8 (corrected)	Paraneoplastic hypercalcemia	Reversible with Ca correction and malignancy treatment

Initial therapy consists of aggressive intravenous hydration with isotonic saline to lower calcium levels and stabilize hemodynamics. For sustained control, intravenous bisphosphonates (such as zoledronic acid) are recommended to inhibit osteoclastic bone resorption, though their onset is delayed. Calcitonin provides a rapid, short-acting reduction in serum calcium and is particularly helpful in symptomatic bradyarrhythmias. In refractory or high-risk cases, denosumab is an established alternative, especially in renal impairment. Hemodialysis remains a life-saving option for patients with severe, treatment-resistant hypercalcemia. Effective recognition and treatment can restore sinus rhythm and improve overall outcomes^[24]^.

This case reinforces several important clinical lessons. Electrolyte disturbances must remain in the differential diagnosis of unexplained bradyarrhythmias. While potassium and magnesium abnormalities are well recognized, hypercalcemia should also be considered, as timely correction can avert unnecessary interventions. Additionally, hypercalcemia with suppressed PTH should not be attributed to renal dysfunction but should instead prompt evaluation for malignancy, particularly when PTHrP is elevated. In this patient, appropriate recognition and treatment of hypercalcemia not only restored sinus rhythm but also improved renal function and systemic symptoms. Although the hypercalcemia in this case may represent a paraneoplastic process, tissue confirmation needs to be done yet.

## Conclusion

Hypercalcemia is an uncommon but important and reversible cause of bradyarrhythmia. While its electrocardiographic manifestations classically include QT interval shortening, rare presentations such as sinus bradycardia or heart block may occur and can be clinically significant. This case illustrates that, in elderly patients, even moderate conduction slowing may cause presyncope and other symptoms, and recognition of hypercalcemia as the underlying etiology is essential. Correction of calcium levels can restore normal sinus rhythm and avert unnecessary pacing interventions. Furthermore, hypercalcemia in the setting of suppressed PTH should not be attributed to renal dysfunction but should instead prompt evaluation for paraneoplastic syndromes, particularly when PTHrP is elevated; however, elevation alone is not diagnostic, and malignancy should not be presumed in the absence of confirmatory imaging or tissue pathology. Awareness of this rare but reversible cause of bradyarrhythmia may improve diagnostic accuracy and patient outcomes.

## Data Availability

Data are available upon request.

## Patient perspective

Patient tolerated the treatment with no adverse effects and good outcomes. He was thankful for the team.

## Research registration unique identifying number (UIN)

Not required for a case report.

## References

[1] Sadiq NM Naganathan S. Hypercalcemia. StatPearls. Treasure Island (FL). StatPearls Publishing; 2024.

[2] Sulaiman S Khan S Ahmad M. Prevalence and etiological profile of hypercalcemia in general medicine wards: a real-world study. Cureus 2022;14:e27853.36110436

[3] Stewart AF. Hypercalcemia associated with cancer. N Engl J Med 2005;352:373–79. 15673803 10.1056/NEJMcp042806

[4] Marcocci C Cetani F. Clinical practice. Primary hyperparathyroidism. N Engl J Med 2011;365:2389–97. 22187986 10.1056/NEJMcp1106636

[5] Shah VN Bhadada SK. Endocrine and metabolic causes of hypercalcemia. Indian J Endocrinol Metab 2012;16:221–27.

[6] Kalla A Figueredo VM. Electrocardiographic manifestations of electrolyte abnormalities. J Electrocardiol 2018;51:828–32.

[7] Iqbal AM Burgess R Gallacher DJ. Electrocardiogram manifestations of hypercalcemia. Cureus 2020;12:e8253.32596072

[8] Singh N Singh HK Khan IA. Hypercalcemia-induced complete heart block: a rare reversible entity. Case Rep Cardiol 2016;2016:2534672.

[9] Evenepoel P Cunningham J Ferrari S. Calcium metabolism and chronic kidney disease. Clin J Am Soc Nephrol 2021;16:806–17.33441463

[10] Mirrakhimov AE. Hypercalcemia of malignancy: an update on pathogenesis and management. N Am J Med Sci 2015;7:483–93. 26713296 10.4103/1947-2714.170600 PMC4683803

[11] Goldner W. Cancer-related hypercalcemia. J Oncol Pract 2016;12:426–32. 27170690 10.1200/JOP.2016.011155

[12] Agha RA Mathew G Rashid R. Transparency in the reporting of Artificial INtelligence–theTITAN Guidelines. Prem J Sci 2025;10:100082.

[13] Singh N Singh H Khan IA. Hypercalcemia-induced complete heart block: a reversible entity. Pacing Clin Electrophysiol 2004;27:555–56.15078416

[14] Hamilton S Terentyev D. ER stress and calcium-dependent arrhythmias. Front Physiol 2022;13:1041940. 36425292 10.3389/fphys.2022.1041940 PMC9679650

[15] Murray SL. Calcium and phosphate disorders: core curriculum 2024. Am J Kidney Dis 2024;83:551–65. 10.1053/j.ajkd.2023.04.017 38099870

[16] Akinseye OA Shahreyar M Heckle MR. A rare case of severe hypercalcemia causing sinus arrest and review of literature. Am J Med Case Rep 2015;3:280–83.

[17] Gutiérrez JA Kancherla D Patel NP. Hypercalcemia-induced complete heart block: a rare manifestation. Tex Heart Inst J 2016;43:527–29.

[18] Ramakumar V Balaram V Suneja M. Complete heart block secondary to hypercalcemia: a case report and literature review. Cureus 2021;13:e13721.33833932

[19] Vosnakidis A Nikas N Soufras GD. Intermittent 2:1 atrioventricular block associated with hypercalcemia due to primary hyperparathyroidism. Hellenic J Cardiol 2013;54:463–67.

[20] Thotakura S Master A Hegde S. Paraneoplastic hypercalcemia presenting with complete heart block. Fed Pract 2016;33:36–39.30766182

[21] Kelwade JV Chawla S Prabhu S. Electrocardiographic manifestations in hypercalcemia: a review of literature. J Assoc Physicians India 2016;64:83–84.

[22] Abu-Abaa M Al-Azzam S. Al-Horani R. Hypercalcemia-induced sinus bradycardia: a reversible presentation. Eur J Case Rep Intern Med 2022;9:003624.36415840

[23] Khederlou H Esmaeili S Fakharian S. Complete heart block in paraneoplastic hypercalcemia: case report and literature review. Eur Heart J Case Rep 2023;7:ytad020. 36694870 10.1093/ehjcr/ytac492 PMC9856270

[24] El-Hajj Fuleihan G Clines GA Hu MI. Treatment of hypercalcemia of malignancy in adults: an Endocrine Society clinical practice guideline. J Clin Endocrinol Metab 2023;108:507–28. 36545746 10.1210/clinem/dgac621

